# Development of a quantitative North and Central European job exposure matrix for wood dust

**DOI:** 10.1093/annweh/wxad021

**Published:** 2023-05-11

**Authors:** Ioannis Basinas, Tuula Liukkonen, Torben Sigsgaard, Nils T Andersen, Jesper M Vestergaard, Karen S Galea, Martie van Tongeren, Ruth Wiggans, Barbara Savary, Wijnand Eduard, Henrik A Kolstad, Anne Vested, Hans Kromhout, Vivi Schlünssen

**Affiliations:** Centre for Occupational and Environmental Health, School of Health Sciences, Faculty of Biology, Medicine and Health, University of Manchester, Manchester Academic Health Science Centre, Manchester, United Kingdom; Institute of Occupational Medicine, Edinburgh, United Kingdom; Department of Public Health, Environment, Occupation and Health, Danish Ramazzini Centre, Aarhus University, Aarhus, Denmark; Finnish Institute of Occupational Health, Helsinki, Finland; Department of Public Health, Environment, Occupation and Health, Danish Ramazzini Centre, Aarhus University, Aarhus, Denmark; Department of Public Health, Environment, Occupation and Health, Danish Ramazzini Centre, Aarhus University, Aarhus, Denmark; Department of Occupational Medicine, Danish Ramazzini Centre, Aarhus University Hospital, Aarhus, Denmark; Institute of Occupational Medicine, Edinburgh, United Kingdom; Centre for Occupational and Environmental Health, School of Health Sciences, Faculty of Biology, Medicine and Health, University of Manchester, Manchester Academic Health Science Centre, Manchester, United Kingdom; Centre for Occupational and Environmental Health, School of Health Sciences, Faculty of Biology, Medicine and Health, University of Manchester, Manchester Academic Health Science Centre, Manchester, United Kingdom; INRS, Centre de Lorraine, Vandoeuvre Les Nancy, France; Department of Chemical and Biological Work Environment, National Institute of Occupational Health, Oslo, Norway; Department of Occupational Medicine, Danish Ramazzini Centre, Aarhus University Hospital, Aarhus, Denmark; Department of Public Health, Environment, Occupation and Health, Danish Ramazzini Centre, Aarhus University, Aarhus, Denmark; Department of Occupational Medicine, Danish Ramazzini Centre, Aarhus University Hospital, Aarhus, Denmark; Institute for Risk Assessment Sciences, Utrecht University, Utrecht, The Netherlands; Department of Public Health, Environment, Occupation and Health, Danish Ramazzini Centre, Aarhus University, Aarhus, Denmark

**Keywords:** exposure database, exposure modelling, historical exposure assessment, time trends, wood dust

## Abstract

Wood dust is an established carcinogen also linked to several non malignant respiratory disorders. A major limitation in research on wood dust and its health effects is the lack of (historical) quantitative estimates of occupational exposure for use in general population-based case-control or cohort studies. The present study aimed to develop a multinational quantitative Job Exposure Matrix (JEM) for wood dust exposure using exposure data from several Northern and Central European countries. For this, an occupational exposure database containing 12653 personal wood dust measurements collected between 1978 and 2007 in Denmark, Finland, France, The Netherlands, Norway, and the United Kingdom (UK) was established. Measurement data were adjusted for differences in inhalable dust sampling efficiency resulting from the use of different dust samplers and analysed using linear mixed effect regression with job codes (ISCO-88) and country treated as random effects. Fixed effects were the year of measurement, the expert assessment of exposure intensity (no, low, and high exposure) for every ISCO-88 job code from an existing wood dust JEM and sampling duration. The results of the models suggest that wood dust exposure has declined annually by approximately 8%. Substantial differences in exposure levels between countries were observed with the highest levels in the United Kingdom and the lowest in Denmark and Norway, albeit with similar job rankings across countries. The jobs with the highest predicted exposure are floor layers and tile setters, wood-products machine operators, and building construction labourers with geometric mean levels for the year 1997 between 1.7 and 1.9 mg/m^3^. The predicted exposure estimates by the model are compared with the results of wood dust measurement data reported in the literature. The model predicted estimates for full-shift exposures were used to develop a time-dependent quantitative JEM for exposure to wood dust that can be used to estimate exposure for participants of general population studies in Northern European countries on the health effects from occupational exposure to wood dust.

## What’s important about this paper?

Previous Job Exposure Matrices (JEMs) for wood dust have been ad hoc and country-specific. The current study describes the empirical modelling underlying the elaboration of a multinational North and Central European quantitative JEM for wood dust. More than 12000 personal measurements collected across size European countries between the period of 1978 and 2007 are used in the process. The JEM will form a valuable tool for assessing historical exposure in large multinational general population-based studies in Europe.

## Introduction

Wood is abundantly used worldwide, and at least 2 million workers in the European Union are employed in the wood manufacturing and furniture industries alone (EUROSTAT 2021). Wood dust originates from the processing and handling of wooden materials. It comprises a complex mixture of particulates of different chemical compositions, which depends on the type of wood being processed. More than 1000 wood species are used for commercial purposes (IARC 1995). The biologically active substances in wood dust, often called “wood extractives,” are high and low molecular weight organic and inorganic compounds with sensitizing and irritant properties. Examples are terpenes and terpene derivatives like plicatic acid, abietic acid, phenolic compounds, tannins, stilbenes, flavonoids, and glycosides (Woods and Calnan 1976). Specific sensitization with IgE binding to single proteins has been demonstrated for, e.g. Western red cedar (Chan-Yeung et al. 1973), pine wood (Skovsted et al. 2003), and obeche wood (Kespohl et al. 2005). Wood dust may also include agents of microbial origin such as endotoxins, glucans, and mycotoxins (Gioffrè et al. 2012).

It is well documented that occupational wood dust exposure can cause sinonasal cancer and evidence also suggests a relationship between occupational wood dust exposure and several cancers of the respiratory and digestive tract (IARC 1995). Wood dust is one of few carcinogens regulated with a binding EU occupational exposure limit (OEL) value, which was recently set to 2 mg/m^3^ for inhalable hardwood dust (https://eur-lex.europa.eu/legal-content/EN/TXT/PDF/?uri=CELEX:32017L2398&from=EN). For softwood dust, OELs remain variable by country ranging between 2 and 5 mg/m^3^ (https://limitvalue.ifa.dguv.de/). Besides cancer, exposure to wood dust can cause asthma (Pérez-Ríos et al. 2010; Wiggans et al. 2016), respiratory symptoms, acute lung function decline and rhino-conjunctivitis (Jacobsen et al. 2010a, 2010b) and is suspected to cause chronic obstructive pulmonary disease (COPD) (Noertjojo et al. 1996; Glindmeyer et al. 2008; Bolund et al. 2018) and interstitial lung disease (ILD) (Gustafson et al. 2007).

A major limitation in research on wood dust and its health effects is the lack of (historical) quantitative estimates of wood dust in population-based case–control or cohort studies. In order to explore rare diseases like ILD, severe COPD or histological subtypes of cancer large-scale population-based studies are needed.

Levels of wood dust exposure vary by country, industrial sector and task/occupation (Vinzents and Laursen 1993; Kauppinen et al. 2006; Schlunssen et al. 2008), with high exposures observed in industries like furniture manufacturing where sanding and other manual wood processing tasks are frequently performed in close proximity to the breathing zone. The variability in average exposure between workers can be large and is generally equal in size to the day-to-day variability within workers for wood-related industries (Scheeper et al. 1995; Vinzents et al 2001). Furthermore, group-based approaches based on tasks were previously shown to result in a reasonably high contrast in exposure (Schlunssen et al. 2004). The use of a Job Exposure Matrix (JEM) for assessing wood dust exposure is therefore appealing. Within the last decade, a framework for calibrating semi-quantitative expert-based JEMs using measurement data has evolved, and this approach has been used to develop a quantitative population-based JEM for benzene (Friesen et al. 2012), population-based JEMs for five carcinogens including silica and asbestos (Peters et al. 2011, 2016), and more recently population-based JEMs for noise (Stokholm et al. 2020) and daytime light exposure (Vested et al. 2019). A comparable approach was also used to develop a quantitative population-based JEM specific for the Canadian population using expert assessments performed for the semi-quantitative CANJEM general population JEM combined with almost 4000 personal and 1500 stationary samples from two provinces in Canada covering the period 1981 to 2003 (Sauvé et al. 2019).

The current study aimed to develop a North and Central European quantitative JEM for wood dust to be used in large multinational general population-based studies. For this, more than 12000 personal measurements from six Northern and Central European countries covering the period between 1978 and 2007 were used and combined with a recently updated expert-assessed JEM (Le Moual et al. 2018) for, among others, wood dust. A second objective of the study was to model long-term temporal trends in personal exposure to wood dust.

## Materials and methods

### Database establishment

An initial exposure database comprising 35201 personal and stationary measurements from Denmark, France, Finland, Norway, the Netherlands, Germany and the United Kingdom (UK) was elaborated. Measurement results from previously performed research and/or already established data information sources were compiled including:

- the German (*n* = 20828), French COLCHIC database (*n* = 7881) (Mater et al. 2022), and Finnish (*n* = 1230) part of the WOODEX database on occupational wood dust exposure and health effects within EU countries between 1987 and 2002, comprising a total of 29939 measurements (Kauppinen et al. 2006);
- the Danish Wood Study performed among furniture workers between 1997 and 2004 included 3572 measurements (Schlunssen et al. 2008);
- a Dutch exposure study of 343 measurements among workers in joineries and furniture factories collected within the years 1992 to 1993 (Scheeper et al. 1995);
- an exposure survey of 41 measurements among Norwegian cabinet workers performed in 1978 as part of a response from the “Yrkeshygienisk Institute” to health complaints from related workers (Johnsen and Pedersen 1978);
- a series of exposure surveys comprising 635 personal measurements in the UK wood industry performed by the Institute of Occupational Medicine (IOM) and the Health and Safety Executive (HSE) between 1985 and 2005 (Black et al. 2007; Galea et al. 2009).
- an exposure survey of 399 personal measurements in the wood and furniture industries performed by the Danish Working Environment Authority (Arbejdstilsynet) in 1988 (Arbejdstilsynet 1989);
- 272 measurements from the exposure databases covering the period following the year 2002 of the Finnish Institute of Occupational Health (FIOH) (Kauppinen 2001).

All measurements were assigned job and industry codes based on the provided process and/or job descriptions. For industries, codes were assigned according to the Danish adaptation of the Statistical Classification of Economic Activities in the European Community (NACE rev 2) (StatisticsDenmark 2015). For job titles, codes were allocated (one to four-digits depending on the accuracy of the job description) according to the ‘International Classification of Occupations (ISCO), 1988 edition (ILO 1990). Coding and data management were performed at an individual data-source level. Data were collated into a common database together with auxiliary information including data source, country, and measurement attributes such as type of measurement, year, duration, sampling fraction and sampling device, and when available task involved, measurement reason and measurement strategy.

## Data curation and management

Following collation, the database contents were restricted to those measurements that were addressing exposure to wood dust, personal measurements, had adequate information on sampling devices used, could represent full shifts, and were collected using an adequate methodology (e.g. not with gas probes, silica gel tubes, or being personal measures collected with high volume dust sampling devices) and could be ISCO-88 coded.

This led to exclusion of measurements that:

- were not personal (*n* = 9255)
- were not wood dust measurements, that is, were either collected through improper methods (*n* = 57) or did not involve exposure to wood dust (e.g. performed during work related to manufacturing and extraction of plastics, welding etc; *n* = 793)
- were missing contextual information regarding the sampling device used, year and type of measurement (i.e. personal or stationary) (*n* = 511)
- did not include sufficient descriptions to be assigned with a job code (*n* = 3509)
- had a sampling time >600 min (*n* = 14) or <60 min (*n* = 290) (Peters et al. 2011) and
- were from the German part of the WOODEX database (*n* = 8,119). The German data comprised measurements obtained from workplaces with expected high wood dust concentrations under an intervention study design—i.e. high concentrations of measured wood dust triggered improvements in the installation of exhaust ventilation with measurements before and after the intervention. Furthermore, the vast majority were short-term measurements (mean (SD) sampling time of 123 (38) min).

These exclusions resulted in 12653 personal measurements from Denmark, Finland, Norway, the Netherlands, France and the United Kingdom remaining available for modelling of the exposure (Table 1).

**Table 1.** Basic characteristics of wood dust measurements included in the final dataset.

Covariates		Dust measurements,
		*n* (%)
*General characteristics*		
Type of measurements	Personal	12653 (100)
Reason for sampling	Survey	4734 (37.4)
	Inspection/compliance	160 (1.3)
	Unknown	7759 (61.3)
Sampling strategy	Representative	12471 (98.6)
	Not representative	182 (1.4)
OAs-JEM score	No exposure	735 (5.8)
	Low exposure	2800 (22.1)
	High exposure	9118 (72.1)
Year of measurement	Year, mean (SD)	1997 (4.7)
Country	Denmark	3719 (29.4)
	Norway	39 (0.3)
	The Netherlands	342 (2.7)
	Finland	642 (5.1)
	United Kingdom	499 (3.9)
	France	7412 (58.6)
*Measurement characteristics*		
Sampling duration	Minutes, mean (SD)	265.9 (108.8)
Type of sampler	Closed-faced cassette	7752 (61.3)
	Open faced cassette	129 (1.0)
	7-Hole sampler	136 (1.1)
	IOM sampler	4636 (36.6)
Measurements < LOD		403 (3.2)

All measurements that were provided represented concentrations measured during the original sampling time and were not standardised for the duration of sampling involved (i.e. calculating time-weighted averages) for the purpose of the analyses. Measurement data were adjusted for differences in inhalable dust sampling efficiency resulting from the use of different dust samplers. Correction factors were extracted from previous field studies comparing the sampling efficiency of different samplers used for sampling wood dust, with the IOM inhalable dust sampler as reference (see Supplementary Table S1). Adjustment factors were applied using the median values estimated for each of the included samplers.

Previous research has demonstrated that for measurements below the limit of quantification (LOQ), imputation methods are generally preferable to substitution (Hewett and Ganser 2007; Flynn 2010; Ogden 2010). For results that were below the LOQ, a single imputation method was used, based on a maximum likelihood estimation method (Lubin et al. 2004), to impute a quantitative exposure level. To account for variations in LOQ levels resulting from differences in sampling durations and sampler heads, the imputations were performed based on the mass of dust collected on the filter (mg). Where no mass for measurements below LOQs was available (1.2%), a LOQ comparable to the lowest realistic measured value within the corresponding source dataset was used. Samples stated as <LOQ with a reported sampled mass of dust exceeding 0.2 mg (*n* = 51) were considered unrealistic and excluded from further analysis. Measurements with unknown sampling duration were assigned the median value (in minutes) of their origin country (i.e. 295, 282, and 252 for Denmark, Finland, and the United Kingdom, respectively).

## Statistical modelling of trends in exposure to wood dust

All statistical analyses were performed on log-transformed exposure concentrations using Statistical Analysis Software v.9.4 (SAS institute, Cary, North Carolina, United States of America). Log transformation was decided on after a visual inspection of the distribution of the available wood dust exposure data. This showed that the distribution of the data was very similar to a lognormal distribution. Modelling of the exposure was performed with linear mixed effect regression using the MIXED procedure. Assigned ISCO-88 codes and country were treated as random effects. Measurement year, measurement duration (in minutes), sampling strategy and/or reason for sampling, and the exposure ratings for wood dust from the recently developed expert-assessed OAsJEM (Occupational Asthma JEM) (Le Moual et al. 2018) were considered fixed effects. Inclusion of the OAsJEM exposure intensity ratings (no, low, or high exposure) allowed for extrapolation of the exposure estimates to occupations/job titles where measurements were not available within the database, as well as to overwrite model results for job titles with measurements which are believed to be unexposed (e.g. chief executives, managers, teachers, and mechanics) (Peters et al. 2011). The year, ISCO-88 code and country were all parameters of the Wood Dust JEM to be established whereas sampling strategy and duration of sampling were included to address potential confounding in time trends and exposure estimates. Since individual measurements were corrected for the presence of systematic variations due to sampler efficiencies (Supplementary Table S1) neither the sampling device nor dust fraction was included in the models. The sampling device was strongly correlated with the country (*r* = −0.9; *P* < 0.001) and thereby not included in the model-building process.

A forward model build approach was followed with measurement year (the reference year 1997) a priori included in the models. Variables were then included sequentially based on the improvement of the model fit by means of Akaike Information Criterion (AIC) values. At the final model building stage, the OAsJEM exposure intensity ratings (three levels: No exposure, low exposure, and high exposure) were included to allow the assignment of exposure levels to exposed ISCO-88 codes not covered by our database. Country effects were modelled as a categorical variable with five categories: Scandinavia (Denmark and Norway), France, Finland, the Netherlands, and the United Kingdom. Danish and Norwegian data were grouped together to accommodate the small number of Norwegian measurements available which though provided important information regarding the exposure levels in a time period prior to the 1980’s. To account for the hierarchical structure of the ISCO-88 classification system and to assign exposure levels to minor-occupational groups the final established models were re-fitted using only the first three-digits of the ISCO-88 job code. A uniform covariance structure was assumed between job codes and a Restricted Maximum Likelihood estimation method was used to estimate variance components. Model adequacy was evaluated through influence and residual diagnostics.

The structure of the final established model was as follows:


$$\begin{array}{l} Ln\left(Y\right)=\beta_{0}+\beta_{y}\times Year+\beta_{t}\times Duration+\beta_{j}\\ \times JEM-score+X_{I}\times ISCO+X_{C}\times Country+\varepsilon \end{array}$$

where, Ln(Y) = the natural long transformed wood dust concentration, β_0_ = the intercept, β_y_ × Year = the effect of the measurement year (in years with 1997 as a reference), β_t_ × Duration = the effect of the sampling duration (in minutes), β_j_ × JEM-score = the effect for the OAsJEM exposure intensity rating (categorical variable with three levels), X_I_ × ISCO = the random effect for job-title (1-31 ISCO-88 unit group codes), X_c_ × Country = the random effect for the country (categorical variable with five levels), and ε = the residual.

The robustness of the derived estimates by the models was examined in a series of sensitivity analyses involving repeating the models after: (i) removing the exposure ratings of the OAsJEM from the fixed effects, (ii) excluding all measurement results above 50 mg/m^3^, and (iii) excluding all measurements with a sampling time <240 min.

## Establishment of the JEM

The derived model results were used to predict 8-h time-weighted average (TWA) wood dust exposure for all ISCO-88 codes and the JEM was elaborated in a stepwise process as follows:

1. For low (e.g. roofers) or high exposed (e.g. cabinet makers) ISCO codes by the OAsJEM with more than five measurements in the database, estimates by year and country were obtained directly using the model based on sampling with the IOM sampler and a duration of 480 min.
2. For low or high exposed ISCO codes by the OAsJEM with less than five measurements in the database (e.g. musical instrument makers) the level of exposure was estimated using the predicted levels by year and country for the exposure rating of the OAsJEM, i.e. the country and year specific mean level for low or high exposure depending on the job title in question.
3. For non exposed ISCO codes by the OAsJEM model predictions were overruled and exposure estimates were set to 0 mg/m^3^ across all time periods and countries.

The approach for assigning exposure estimates was identical for both job codes at the minor unit (three-digits) and unit (four-digits) level. Exceptionally, for “Forestry workers and loggers” (ISCO code 6141) for which no measurements were available, an exposure level equivalent to the one predicted for “Forestry Labourers” (ISCO code 9212) was assigned, based on the similarity of the activities performed by the two groups.

## Results

**Fig. 1.**
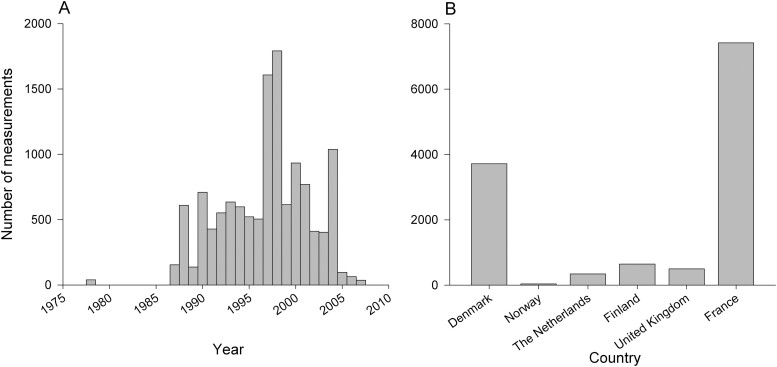
Distribution of measurements in the database across years (A) and countries (B).

**Fig. 2.**
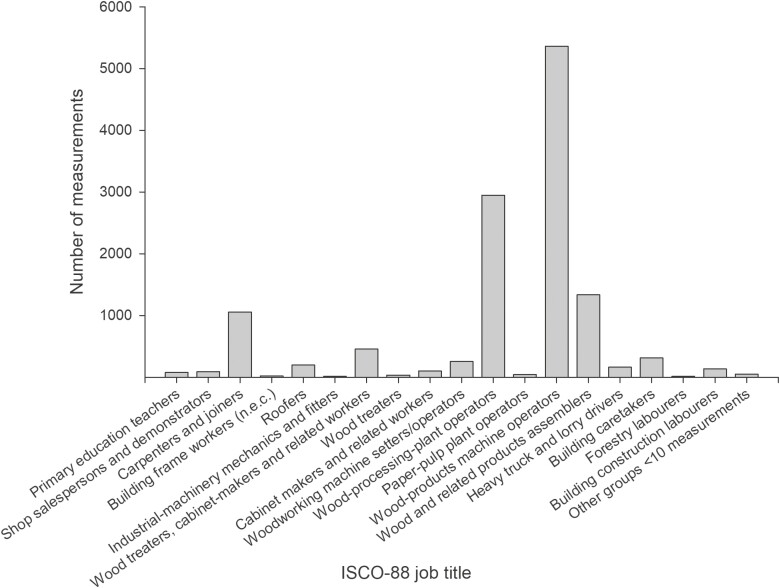
Distribution of measurements in the database across job-titles as defined by the International Classification of Occupations (ISCO), 1988 edition.

Table 1 summarises the attributes of the final dataset comprising 12653 personal wood dust measurements. The included measurements were collected between 1978 and 2007, had an average sampling time of 4.4 h and were mostly (88%) from France and Denmark (Fig. 1). Overall, measurements for 31 job -codes at the ISCO-88 unit group level (four-digits) belonging to 23 job-codes at the ISCO-88 minor group level (three-digits) were included within the established exposure database, covering 15 of the 18 job-codes considered as exposed in the OAsJEM. Most of the included measurements (72.1%) were collected from jobs classified as highly exposed by the OAsJEM such as “wood treaters, cabinet-makers and related trades workers” (ISCO-88 code: 7420), “wood-processing-plant operators” (ISCO-88 code: 8141), and “wood-products machine operators” (ISCO-88 code: 8240) (Fig. 2).

Table 2 provides information on the estimated annual trends and model fit for the various stages in the model development, together with the estimated variance components. When only year was included in the model there appeared to be a downward trend in the exposure levels by almost 9% per year. After adjustment for country and sampling durations, the estimated annual trends in exposure were reduced to 7.8% (*P* < .0001). The inclusion of the expert-based exposure intensity ratings from the AOsJEM neither improved the fit of the model nor changed the estimated trends in an exposure. The final model explained approximately 22.5% of the total variance in the exposure data and reduced the within-country variance by 40%, the between-job (ISCO-code) variance by 59%, and the residual variance by 15%.

**Table 2.** Results from linear mixed effect regression describing estimated temporal trends in wood dust exposure in the database. Results are based on 12653 personal wood dust measurement collected between 1978 and 2007.

Model	*β* Year (ref 1997	*e*	*P*-value	Annual trend (%)	AIC	BIC	bcountry σ^2^	biscoσ^2^	resσ^2^
Wood dust									
Naïve					38630.9	38635.2	0.301	0.109	1.232
+ year^*^	−0.094	0.002	<.0001	−9.0	36931.2	36934.1	0.278	0.054	1.078
+ year, sampling duration	−0.081	0.002	<.0001	−7.8	36600.3	36599.1	0.182	0.040	1.048
+ year, sampling duration, AOsJEM score	−0.081	0.002	<.0001	−7.8	36604.8	36603.6	0.182	0.045	1.048

*β =* regression coefficient for log-transformed exposure data; *e =* standard error; *P* = *P*-value; annual trend = % of change in exposure per year estimated as 100*(exp(*β*) − 1); AIC = Akaike Information Criterion; BIC = Bayesian Information Criterion; _bcountry_*σ*^2^ = between country variance; _bisco_*σ*^2^ = between job variance; _res_*σ*^2^ = residual variance. Naïve estimates are derived from a model with random effects (ISCO-88 codes and country) but without fixed effects. * = reference is year 1997.

The parameter estimates for the fixed effects of the final model are shown in Table 3. Besides year, measured concentrations also declined with increased sampling duration by 0.02% per minute of sampling. The results showed considerable differences in exposure between countries with Scandinavian measurements being on average 2 to 3 times lower compared to those measurements collected in other countries. Exposures were highest in the UK and on average there were 20% higher than in France. There was no statistical difference in levels of exposure between jobs classified as no, low, or high exposure by the AOsJEM intensity ratings.

**Table 3.** Linear mixed effect results describing the relationships between log-transformed wood dust levels and fixed effects. Results are based in 12653 measurements collected between 1978 and 2007.

Parameter	*β*	*e*	*P*-value	GMR	95% CI
*Fixed effects*					
Intercept	1.294	0.218	<0.001	4.35	3.57 to 5.31
Year (ref 1997)	−0.081	0.002	<0.001	0.92	0.92 to 0.93
Sampling duration, min	−0.002	0.0001	<0.001	0.89^*^	0.88 to 0.90^*^
OAsJEM score					
No exposure	−0.033	0.133	0.8	0.97	0.75 to 1.26
Low exposure	0.037	0.134	0.8	1.04	0.80 to 1.35
High exposure	Ref			Ref	
*Random effects*					
Country^#^					
DK + NO	−0.733	0.1925	<0.001	0.48	0.33 to 0.70
NL	0.0488	0.1980	0.8	1.05	0.71 to 1.54
FI	0.1445	0.1949	0.5	1.16	0.79 to 1.69
United Kingdom	0.3623	0.1954	0.06	1.43	0.98 to 2.11
FR	0.1771	0.1922	0.3	1.19	0.82 to 1.74
Between-country variance (naive estimate)	0.182 (0.301)	0.13	0.08		
Between-ISCO variance (naive estimate)	0.045 (0.109)	0.019	<0.01		
Residual variance (naïve estimate^^^)	1.04 (1.09)	0.013	<0.0001		
*% of explained variance by the model*					
Between-country variance	39.4				
Between-ISCO variance	58.6				
Residual variance	14.9				
Total variance	22.3				

*β* = beta for log-transformed exposure levels, *e* = standard error, GMR = geometric mean ratio; 95% CI = confidence intervals for the estimated GMR, ISCO = International Standard Classification of Occupations 1988 edition (ISCO-88).

^*^For an increase of 60 min in sampling time.

^^^naïve estimates are derived from a model without fixed factors.

^#^Entered as a random effect in the models, BLUP estimates shown.

Table 4 summarises the predicted levels of wood dust exposure in 1997 based on sampling with the IOM sampler for a duration of a complete working shift (i.e. 480 min) for the five highest and five lowest exposed job titles. The corresponding predicted levels for the AOsJEM intensity ratings for the same year and duration were 0, 0.69 and 0.66 mg/m^3^ of wood dust for the no, low, and high exposure ratings, respectively. The values assigned to the JEM for the year 1997 for all exposed ISCO-88 codes are provided in the online supplement. The highest exposure levels predicted by the model were for floor layers and tile setters (GM 1.92 mg/m^3^), wood-product machine operators (GM 1.78 mg/m^3^), and labourers in construction (GM 1.80 mg/m^3^). Predictions were lowest for the job titles of wood-processing- and handicraft workers in wood, textile, leather, and related materials (GM 1.04 mg/m^3^) followed by wood-processing-plant operators (GM 1.04 mg/m^3^) and wood-processing plant operators (GM 1.10 mg/m^3^).

**Table 4.** Predicted levels of wood dust exposure for the reference year (1997) for the 5 highest and lowest exposed job-codes.

ISCO -88 code	ISCO-88 standard description	Wood dust GM level (mg/m^3^)	
		Non country specific estimate	Range of country-specific estimates
*Highest exposed codes*			
7132	Floor layers and tile setters	1.92	0.92 to 2.76
9313	Building construction labourers	1.80	0.86 to 2.58
8240	Wood-products machine operators	1.78	0.76 to 2.21
7131	Roofers	1.77	0.85 to 2.54
7420	Wood treaters, cabinet-makers, and related trades workers	1.65	0.82 to 2.38
Lowest exposed codes			
9212	Forestry labourers	1.30	0.62 to 1.87
7423	Woodworking machine setters and setter-operators	1.19	0.55 to 1.66
8141	Wood-processing-plant operators	1.10	0.53 to 1.57
7330	Handicraft workers in wood, textile, leather, and related materials	1.04	0.52 to 1.45
8140	Wood-processing- and papermaking-plant operators	1.04	0.52 to 1.45

Sensitivity analysis by removing the exposure ratings of the OAsJEM from the fixed effects, excluding all measured exposure concentrations above 50 mg/m^3^ or with a sampling time < 240 min did not systematically change the results. The predicted values by the final elaborated models in the main analysis and the predicted values from each of the sensitivity analyses described were nearly identical (not shown).

## Discussion

The present paper summarises the development of a quantitative North and Central European JEM for assessing wood dust exposure in a general population. This was achieved using empirical statistical modelling of a large exposure database established for the purpose. Data included more than 12000 cross-industry measurements collected through personal monitoring of woodworkers mostly working between the period 1987 and 2007 in six European countries. Potential determinants that could influence exposure estimates including the year of sampling, sampling duration, the efficiency of the sampling device used, and sampling strategy, were taken into account in an approach comparable to the one previously established within the SYNERGY project (Peters et al. 2011). The developed JEM built on a yearly time scale can be used to, retrospectively, estimate exposure within national and multinational general population studies investigating health risks from occupational exposure to wood dust.

Our modelling results suggest that personal exposures to wood dust have declined annually by almost 8% in the period for which data were available; resulting in an 11-fold reduction in personal exposure to wood dust over the three decades covered by the database. This reduction, likely a result of changes in processes including improvements in technology and legislation, is in concordance with the literature. Coble et al. (2001) analysed trends from compliance measurement data collected in US pulp and paper manufacturing facilities and reported an annual decline of 6% between 1979 and 1997. Teschke et al. (1999) reported “total” wood dust exposure among US workplaces to decrease by a factor of 30 in a 20-yr period from 4.59 mg/m^3^ in 1979 to 0.14 mg/m^3^ in 1997. Similar findings were reported for UK workplaces for the period between 1976 and 1983 (Jones and Smith 1986). Annual declines in the latter two studies were estimated to be in the range of 10% to 11% per annum (Creely et al. 2007). In a more recent analysis, Galea et al. (2009) used more than 1400 measurements (partly overlapping with the present study) to demonstrate an average annual decline of 8.1% in UK workplaces between 1985 and 2005.

Our model suggests that floor layers and tile setters are the highest exposed group of workers to wood dust with an estimated GM level of exposure for the reference year (1997) of 1.9 mg/m^3^, reducing to a GM level of 1.0 mg/m^3^ for the year 2005. This is in line with the results of Scarselli et al. (2008) who, based on 56 measurements collected between 1996 and 2006, reported a GM exposure level for floor layers and tile settlers of 1.1 mg/m^3^. It is worth noting that so far only a few measurements, other than those included in the current database, have been reported for this occupational group in the literature.

Wood-product machine operators and wood treaters, cabinet workers and related trades were estimated by our JEM to be exposed to GM levels of 1.8 and 1.7 mg/m^3^ in the year 1997. These are jobs that cover a wide range of tasks including sanding, planning, sawing, and cutting, and occur in various sectors such as the furniture industry and sawmills. Kalliny et al. (2008), in a survey among 10 wood US processing plants performed between 1999 and 2004, reported a GM for inhalable dust of 2.4 mg/m^3^ for sanding operations in both the furniture (620 measurements) and wood processing (374 measurements) sectors. For sawing the corresponding GMs for the furniture and wood processing industries were 1.7 mg/m^3^ and 1.5 mg/m^3^ of inhalable dust based on 195 and 407 measurements, respectively. Other studies have reported high levels of personal exposure to wood dust during such activities. Gioffrè et al. (2012) in a study involving personal monitoring performed during the late 2010s in four carpentries of Southern Italy, reported the exposure levels of inhalable wood dust among workers in sanding stations to average between 1.75 and 11.28 mg/m^3^. Similarly, Teschke et al. (1999) analysed more than 1600 measurements of wood dust from the US OSHA’s Integrated Management Information System collected in the period between 1979 and 1997. Levels of exposure among sanders in wood cabinet and furniture manufacturing were found to average (GM) between 3.96 and 5.83 mg/m^3^ (Teschke et al. 1999).

We found high exposure levels also for building construction labourers and roofers, with a GM for the reference year of 1.8 mg/m^3^. Few measurements apart from those included in our database are available for construction workers, but the presence of high levels of exposure in construction is generally supported by more recent measurements performed among carpenters on UK building sites (Stacey et al. 2019). It is important to note that workers in construction sites, including carpenters and labourers, are unlikely to be exposed to dust that is solely composed of wood. The previously mentioned study by Stacey et al. reported that the median proportion of minerals in the mass of 29 personal inhalable dust samples collected from carpenters, shopfitters and plumbers was 30% (range: 0% to 62%) (Stacey et al. 2019).

Forestry labourers and wood-processing and papermaking-plant operators were, according to our model, among the lowest exposed with GM levels between 1.3 and 1 mg/m^3^, respectively, for the reference year which correspond to levels well below 1 mg/m^3^ for the year 2000. These findings agree with those from the US wood processing industry where measurements among debarkers collected between 1999 and 2004 averaged (GM) at 1.1 mg/m^3^ (Kalliny et al. 2008). Forestry and sawmill workers are suggested to have mean exposures well below 1 mg/m^3^ as reported across several different country settings (Douwes et al. 2000; Friesen et al. 2006; Straumfors et al. 2018). Similarly, a GM of 0.3 mg/m^3^ was reported for Swedish pulp- and paper-mill workers in the period between 2007 and 2009 (Westberg et al. 2016).

Our model results suggest considerable differences in exposure between countries with the highest exposures being observed among UK workers. Such differences have previously been reported for other agents (de Vocht et al. 2006; Liu et al. 2011; Peters et al. 2011) and could reflect several reasons including differences in regulation (i.e. OEL values) over time, differences in sampling strategies and/or actual large variations in production and working practices between workers. Their presence implies future efforts to apply our Wood Dust JEM to other populations not represented in the underlying database should be done cautiously and after careful review of the working production and process similarities and differences across the countries involved. Comparisons between our JEM with other JEMs that include estimates of wood dust exposure are challenging due to methodological differences in the developments of these JEMs. The Finnish job-exposure matrix (FINJEM) estimates that for cabinet makers, joiners, and floor layers the wood dust exposure is between 0.5 and 1.0 mg/m^3^ (Siew et al. 2012), which is much lower than estimates based on our empirical models (Table 4). In contrast, for the same period, woodworking machine operators are estimated by FINJEM to be exposed to levels of wood dust averaging 2.5 mg/m^3^ which is much closer to our estimates.

In a more recent and comparable effort, Sauvé et al. (2019) developed a quantitative JEM based on Bayesian modelling approaches and 5170 personal and stationary wood dust measurements collected from Canadian workplaces between 1981 and 2003 across 31 occupations rated as exposed by CANJEM. Although very specific to the Canadian working population and coded according to the Canadian National Occupation Classification for Statistics this JEM, like ours, highlights cabinet makers, woodworking machine operators, and floor covering installers as being among the highest exposed occupations with their estimated levels of exposure for the reference year (i.e. 1989) being close to or above 1.5 mg/m^3^. Our across-country estimates for the corresponding jobs of cabinet makers, wood-product machine operators and floor layers were somewhat higher at 2.5, 3.5 and 3.7 mg/m^3^ of wood dust, respectively.

Our models explained more than 55% of the variance between occupations and more than 22% of the total variance in an exposure. This is in line and even better than seen in earlier modelling efforts for the development of quantitative JEMs for agents such as noise (Stokholm et al. 2020), asbestos, nickel, and respirable crystalline silica (Peters et al. 2011, 2016). Yet, most of the variance in exposure within our dataset was allocated in the residual and within countries components. The residual variance component includes differences in exposure between companies, between workers within a job in a company, and day-to-day variability in exposure concentrations. To reduce the residual variance detailed information on individual companies, workers, and on related exposure-affecting factors (e.g. ventilation, process, etc) will be needed. Country differences could reflect variations in production, risk reduction measures, and working practices. This kind of data was not available within our database, which mostly comprised of data collected and curated as part of WOODEX (Kauppinen et al. 2006).

The exclusive use of personal measurements and the substantial number of measurements underlying our modelling process form major strengths of our JEM. Similarly, the multinational nature of our database and, our consequent ability to provide estimates for five different countries/regions covering North and Central Europe, further increase the potential applicability of our JEM in epidemiological studies either examining or adjusting exposure–response relationships for the effects of exposure to wood dust. However, it has to be mentioned that most of the measurements included in the JEM originated from two Countries, Denmark and France, but with a similar job ranking across measurements from all countries. Limitations of our work include the lack of detailed contextual information for the measurement data concerning the type of wood dust involved in the measurements and factors that may affect exposure in the workplace including between workers performing the job (e.g. use of control measures such as local exhaust ventilation). Detailed contextual data, if available, could have further improved the performance of our model, potentially even explaining some of the observed between country differences; however, such information is seldom, if ever, available within general population epidemiological studies for which exposure is mainly established based on job histories. Health effects resulting from wood dust exposure are known to be dependent on the type of wood dust involved (e.g. hardwood, softwood, and bark, etc.) which differs both between within industries and/or jobs (e.g. furniture making entails use of different types of wood over time with solid timber used in early periods substituted later by reconstituted wood such as chipboard). Such differences are important to be considered when interpreting the exposure estimates of our JEM as well as results from any exposure-response analyses using the JEM estimates.

In addition, to account for differences in concentrations caused by different sampling heads with different sampling performances and to smoothen the interpretation of time trends amid evident dependencies in the use of sampling devices across time and countries (not shown), we adjusted our measurement results using published wood dust specific correction factors. The variability in these extracted correction factors is generally large and may be affected by particle distribution and concentration (Tatum et al. 2001; Harper and Muller 2002). For example, differences in the distance of the measurement to the source of exposure can result in larger particles settling at greater distances from the source which also may lead to lower exposure levels (Kromhout et al. 2005). Unfortunately, no information related to distance from the source was available in our database.

Similarly, our measurement database does not include measurements for all the exposed job titles in OAsJEM for the complete period covered in the database. In fact, coverage of measurements for job titles differed also between countries. Yet, we had measurements for more than 80% of the exposed job codes within OAsJEM, which is in line with previous JEMs using comparable approaches (Peters et al. 2011). Perhaps unexpectedly, average exposure levels were somewhat higher among expert-assessed low exposure jobs compared to high-exposed jobs, though this did not reach significance (Table 3). Similar results have also been reported in the analyses carried out for the development of the SYNERGY (Peters et al. 2016) and Canadian Wood (Sauvé et al. 2019) JEMs. A possible explanation could be that measurements for the highly exposed jobs were more likely to be performed during fairly representative conditions. In contrast, for low-exposed jobs the measurements are more likely to have been carried out during specific and relatively infrequent activities involving the use or processing of wood (Peters et al. 2016; Sauvé et al. 2019). However, high and low-exposed jobs were both shown to have higher mean levels of exposure to wood dust compared to the non exposed jobs. Validation exercises of the OAsJEM for wood, or any other agent, are yet to be performed; however, wood dust is relatively easy to link to specific jobs. In an earlier study comparing the performance of two general population JEMs across 25 different exposures, the congruence between the JEMs was highest for wood dust (Kromhout et al. 1992). Excluding the OAsJEM exposure ratings from the modelling process had a negligible effect on the model predicted values. The exposure ratings were kept in the final models so that the model could be used to provide exposure estimates for jobs with few or no measurements (Ramachandran 2001).

## Conclusions

Based on more than 12500 historical personal measurements from six European countries, albeit mostly from Denmark and France, and an empirical modelling approach we developed a quantitative JEM for wood dust exposure that can be used to assign wood dust exposure to population-based studies with information on specific occupations for the period between 1978 and 2007. The derived exposure estimates are plausible and comparable with the wood dust levels reported in the literature for the corresponding jobs and period of time. However, large differences in exposure between countries were observed, which could reflect differences in production, risk reduction measures, and working practices. Average exposure levels have declined by almost 8% per year within the period with available measurement data resulting in an 11-fold reduction over the three decades covered. The established JEM can be used to provide wood dust exposure estimates for national and multinational general population case-control and cohort studies in the northern and central European countries covered by the JEM. For other countries, the JEM should only be used with caution. It is anticipated that its quantitative nature and geographical coverage will enhance the ability of such studies in Europe to evaluate existing exposure–response relationships between exposure to wood dust and related health effects.

## Supplementary Material

wxad021_suppl_Supplementary_Material
