# Supplementary material for: Development of a quantitative North and Central European job exposure matrix for wood dust
Source: Ann Work Expo Health. 2023 May 11;67(6):758–71. doi: 10.1093/annweh/wxad021 (PMC10795000; doi:10.1093/annweh/wxad021)
Supplement: wxad021_suppl_Supplementary_Material [file wxad021_suppl_supplementary_material.docx]

**Online Supplementary Table**

**Development of a Quantitative North and Central European Job Exposure Matrix for Wood Dust.**

Ioannis Basinas,^1;2;3^ Tuula Liukkonen,^4^ Torben Sigsgaard,^3^ Nils T. Andersen,^3^ Jesper M. Vestergaard,^5^ Karen Galea,^2^ Martie van Tongeren,^1^ Ruth Wiggans,^6^ Barbara Savary,^7^ Wijnand Eduard,^8^ Henrik A. Kolstad^,5^ Anne Vested,^4;5^ Hans Kromhout,^9‡^ Vivi Schlünssen.^3^ ^‡^.

*^1^ Centre for Occupational and Environmental Health, School of Health Sciences, Faculty of Biology, Medicine and Health, University of Manchester, Manchester Academic Health Science Centre, Manchester, United Kingdom*

*^2^ Institute of Occupational Medicine, Edinburgh, United Kingdom*

*^3^ Department of Public Health, Environment, Occupation and Health, Danish Ramazzini Centre, Aarhus University, Aarhus, Denmark*

*^4^Finnish Institute of Occupational Health, Helsinki, Finland;*

*^5^ Department of Occupational Medicine, Danish Ramazzini Centre, Aarhus University Hospital, Aarhus, Denmark*

*^6^ Health & Safety Laboratory Buxton, Derbyshire, United Kingdom*

*^7^INRS, Centre de Lorraine, Vandoeuvre Les Nancy, France*

*^8^ Department of Chemical and biological work environment, National Institute of Occupational Health, Oslo, Norway*

*^9^ Institute for Risk Assessment Sciences, Utrecht University, Utrecht, the Netherlands*

*‡Shared last co-authorship*

**Table S1** Literature reported relative efficiencies of different samplers used for sampling of wood dusts. Results are summarised with the IOM sampler as reference.

| **Type of sampler** | **Correction factor** | | | | | | | | | |
| --- | --- | --- | --- | --- | --- | --- | --- | --- | --- | --- |
|  | Kromhout et al. (2005); n=16 | Tatum et al. (2001); n=23^a^ | Liden et al. (2000); n=173 | Kauffer et al. (2010); n=12 | Vaughan et al. (1990); n=12 | Werner et al. (1996); n=50 | Schlunssen et al. (2001); n=106 | Harper and Muller (2002); n=16 | Martin and Zalk (1998); n=25 | **Median** |
| **IOM** | 1 | 1 | 1 | 1 | 1 | 1 | 1 | 1 | 1 | **1** |
| **Millipore closed faced** | 2.00 | 1.85^b^ |  | 2.02^b^ |  | 1.79^b;^  1.79^c^ | 1.59 | 3.35 ^ab^ | 5.5 | **1.92** |
| **Millipore open faced** | 2.21 |  | 2.5 |  |  |  |  |  |  | **2.36** |
| **GSP** |  | 1.27 |  |  |  |  | 1.01 |  |  | **1.14** |
| **7 hole sampler** |  | 1.38 |  |  | 1.28^d^  1.36^e^ |  |  |  |  | **1.36** |

n=number of pair measurements unless otherwise stated; a = in runs of 6 pair samples; b=4mm orifice; c=5.6 mm orifice; c=Casella 7-hole; d=J.S. Holdings 7-hole;

**Table S2.** Estimated level of personal exposure to wood dust and related 95% confidence intervals (in parenthesis) for all exposed jobs for the year 1997. Country specific and non-country specific estimates are shown.

| **ISCO88** | **jobcode description** | **n of measurements included in final database** | **Wood dust exposure level (mg/m^3^) in 1997** | | | | | |
| --- | --- | --- | --- | --- | --- | --- | --- | --- |
|  |  |  | **Non-country specific exposure estimate** | **DK and NO country specific estimates** | **NL country specific estimates** | **FI country specific estimates** | **UK country specific estimates** | **FR country specific estimates** |
| 6141 | Forestry workers and loggers | 0 | 1.30 (0.78-2.17) | 0.62 (0.44-0.88) | 1.36 (0.94-1.96) | 1.50 (1.05-2.15) | 1.87 (1.30-2.68) | 1.55 (1.09-2.20) |
| 7124 | Carpenters and joiners | 1056 | 1.64 (1.12-2.40) | 0.79 (0.73-0.86) | 1.72 (1.51-1.95) | 1.90 (1.69-2.13) | 2.36 (2.10-2.66) | 1.96 (1.81-2.12) |
| 7129 | Building frame and related trades workers not elsewhere classified | 20 | 1.41 (0.86-2.31) | 0.68 (0.49-0.94) | 1.48 (1.05-2.08) | 1.63 (1.17-2.28) | 2.03 (1.45-2.84) | 1.68 (1.21-2.32) |
| 7131 | Roofers | 196 | 1.77 (1.18-2.65) | 0.85 (0.73-0.99) | 1.86 (1.56-2.22) | 2.05 (1.73-2.43) | 2.54 (2.14-3.02) | 2.11 (1.83-2.44) |
| 7132 | Floor layers and tile setters | 8 | 1.92 (1.12-3.29) | 0.92 (0.63-1.35) | 2.02 (1.36-3.01) | 2.22 (1.50-3.30) | 2.76 (1.86-4.10) | 2.29 (1.56-3.37) |
| 7312 | Musical instrument makers and tuners | 3 | 1.54 (1.02-2.34) | 0.74 (0.62-0.89) | 1.62 (1.31-2.00) | 1.78 (1.45-2.18) | 2.21 (1.80-2.71) | 1.84 (1.53-2.21) |
| 7330 | Handicraft workers in wood, textile, leather and related materials | 0 | 1.04 (0.68-1.60) | 0.50 (0.40-0.62) | 0.52 (0.41-0.66) | 1.10 (0.88-1.38) | 1.45 (1.15-1.83) | 1.26 (1.02-1.56) |
| 7331 | Handicraft workers in wood and related materials | 0 | 1.54 (1.02-2.34) | 0.74 (0.62-0.89) | 1.62 (1.31-2.00) | 1.78 (1.45-2.18) | 2.21 (1.80-2.71) | 1.84 (1.53-2.21) |
| 7420 | Wood treaters, cabinet-makers and related trades workers | 455 | 1.65 (1.13-2.42) | 0.82 (0.75-0.89) | 1.80 (1.58-2.05) | 2.03 (1.81-2.28) | 2.38 (2.12-2.68) | 2.06 (1.89-2.247) |
| 7421 | Wood treaters | 35 | 1.39 (0.87-2.21) | 0.67 (0.51-0.89) | 1.46 (1.08-1.97) | 1.60 (1.20-2.14) | 1.99 (1.51-2.63) | 1.66 (1.26-2.19) |
| 7422 | Cabinet makers and related workers | 100 | 1.33 (0.87-2.03) | 0.64 (0.53-0.77) | 1.39 (1.12-1.72) | 1.53 (1.24-1.89) | 1.90 (1.54-2.35) | 1.58 (1.30-1.92) |
| 7423 | Woodworking machine setters and setter-operators | 254 | 1.19 (0.80-1.77) | 0.57 (0.50-0.65) | 1.25 (1.06-1.48) | 1.37 (1.17-1.60) | 1.71 (1.47-1.99) | 1.42 (1.25-1.62) |
| 8140 | Wood-processing- and papermaking-plant operators | 2997 | 1.04 (0.68-1.60) | 0.49 (0.39-0.61) | 1.08 (0.85-1.37) | 1.21 (0.96-1.52) | 1.43 (1.13-1.80) | 1.24 (1.00-1.54) |
| 8141 | Wood-processing-plant operators | 2948 | 1.10 (0.75-1.61) | 0.53 (0.49-0.57) | 1.15 (1.02-1.30) | 1.27 (1.15-1.40) | 1.57 (1.41-1.75) | 1.31 (1.23-1.39) |
| 8240 | Wood-products machine operators | 5361 | 1.78 (1.22-2.60) | 0.76 (0.72-0.80) | 1.67 (1.49-1.87) | 1.88 (1.70-2.08) | 2.21 (1.98-2.46) | 1.92 (1.81-2.04) |
| 8285 | Wood and related products assemblers | 1334 | 1.31 (0.89-1.92) | 0.63 (0.59-0.67) | 1.37 (1.22-1.54) | 1.51 (1.34-1.70) | 1.88 (1.66-2.13) | 1.56 (1.43-1.70) |
| 9212 | Forestry labourers | 14 | 1.30 (0.78-2.17) | 0.62 (0.44-0.88) | 1.36 (0.94-1.96) | 1.50 (1.05-2.15) | 1.87 (1.30-2.68) | 1.55 (1.09-2.20) |
| 9313 | Building construction labourers | 134 | 1.80 (1.19-2.72) | 0.86 (0.72-1.02) | 1.89 (1.55-2.30) | 2.08 (1.72-2.52) | 2.58 (2.12-3.13) | 2.14 (1.81-2.53) |

**References**

Harper M, Muller BS. (2002) An evaluation of total and inhalable samplers for the collection of wood dust in three wood products industries. J Environ Monit; 4: 648-56.

Kauffer E, Wrobel R, Gorner P, Rott C, Grzebyk M, Simon X, Witschger O. (2010) Site comparison of selected aerosol samplers in the wood industry. Ann Occup Hyg; 54: 188-203.

Kromhout H, Witschger O, Koch W. (2005) In situ testing of a calibration tool for workplace aerosol samplers (CALTOOL). Book In situ testing of a calibration tool for workplace aerosol samplers (CALTOOL), City.

Liden G, Melin B, Lidblom A, Lindberg K, Noren JO. (2000) Personal sampling in parallel with open-face filter cassettes and IOM samplers for inhalable dust--implications for occupational exposure limits. Appl Occup Environ Hyg; 15: 263-76.

Martin JR, Zalk DM. (1998) Comparison of Total Dust/Inhalable Dust Sampling Methods for the Evaluation of Airborne Wood Dust. Appl Occup Environ Hyg; 13: 177-82.

Schlunssen V, Vinzents PS, Mikkelsen AB, Schaumburg I. (2001) Wood dust exposure in the Danish furniture industry using conventional and passive monitors. Ann Occup Hyg; 45: 157-64.

Tatum VL, Ray AE, Rovell-Rixx DC. (2001) The performance of personal inhalable dust samplers in wood-products industry facilities. Appl Occup Environ Hyg; 16: 763-9.

Vaughan NP, Chalmers CP, Botham RA. (1990) Field comparison of personal samplers for inhalable dust. Ann Occup Hyg; 34: 553-73.

Werner MA, Spear TM, Vincent JH. (1996) Investigation into the impact of introducing workplace aerosol standards based on the inhalable fraction. Analyst; 121: 1207-14.
